# The school environment in the experience of children with special health needs: a qualitative study

**DOI:** 10.1590/1980-220X-REEUSP-2024-0215en

**Published:** 2025-01-27

**Authors:** Álida Maria de Oliveira Andreato, Eliane Tatsch Neves, Maiara Rodrigues dos Santos, Maria Angélica Marcheti, Maira Deguer Misko

**Affiliations:** 1 Universidade Estadual de Campinas, Faculdade de Enfermagem, Campinas, SP, Brazil.; 2 Universidade Federal de Santa Maria, Santa Maria, RS, Brazil.; 3 Universidade de São Paulo, Escola de Enfermagem, São Paulo, SP, Brazil.; 4 Universidade Federal de Mato Grosso do Sul, Campo Grande, MS, Brazil.

**Keywords:** Disabled Children, Mainstreaming, Education, Pediatric Nursing, Chronic Disease, Symbolic Interactionism, Niños con Discapacidad, Integración Escolar, Enfermería Pediátrica, Enfermedad Crónica, Interaccionismo Simbólico

## Abstract

**Objective:**

To understand the experience of children with special health needs at school.

**Method:**

Qualitative research using Symbolic Interactionism as a theoretical framework and assumptions of Grounded Theory as a methodological framework. Data collected in a pediatric outpatient clinic of a teaching hospital in an inland city of the state of São Paulo. The following collection strategies were used: participant observation, drawing, and semi-structured interviews. Twenty-two school-age children with special health needs participated in the research, all of whom attended school.

**Results:**

Three categories were constructed representing the experience of children with special health needs at school: Experiencing the school environment, Identifying obstacles, and Finding ways to move forward despite the illness. These children’s experiences at school allowed us to understand their difficulties, their need for adaptation and their actions in the face of the process related to their development.

**Conclusion:**

The need for acceptance, safety, and trust in the school environment and in the people who make it up was identified, which are fundamental factors for the children to be able to continue their development and learning, despite the illness.

## RESUMEN

**Objetivo:**

Comprender la experiencia de los niños con necesidades especiales de salud en la escuela.

**Método:**

Investigación cualitativa que utilizó el Interaccionismo Simbólico como marco teórico y los supuestos de la Teoría Fundamentada como marco metodológico. Datos recolectados en un ambulatorio pediátrico de un hospital universitario del interior del estado de São Paulo. Se utilizaron las siguientes estrategias de recolección: observación participante, dibujo y entrevistas semiestructuradas. En la investigación participaron 22 niños en edad escolar con necesidades especiales de salud, todos asistiendo a la escuela.

**Resultados:**

Se crearon tres categorías que representan la experiencia de los niños con necesidades especiales de salud en la escuela: Experimentar el ambiente escolar, identificar obstáculos y encontrar formas de continuar a pesar de la enfermedad. Las vivencias de estos niños en la escuela nos permitieron comprender sus dificultades, su necesidad de adaptación y sus acciones en relación al proceso relacionado con su desarrollo.

**Conclusión:**

Se identificó la necesidad de aceptación, seguridad y confianza en el ambiente escolar y en las personas que lo integran, los cuales son factores fundamentales para que el niño pueda continuar con su desarrollo y aprendizaje, a pesar de la enfermedad.

## INTRODUCTION

Technological and scientific improvements, especially in the health area, have reduced infant mortality rate in Brazil, leading to an epidemiological change, marked by an increase in survival rates for clinically fragile children. Therefore, health care policies were required aimed at this group that demands continuous care from health services, called Children with Special Health Needs (*Crianes*), which includes children with chronic conditions^(1)^.

In 2022, the first epidemiological survey of the prevalence of *Crianes* in Brazil was carried out, revealing that one in four families has a child aged zero to 11 with special health needs. This datum is associated with the child’s previous health circumstances, family structure, and socioeconomic and political context^(2)^.

The *Crianes* live in the social spaces of daycare centers and schools, which play an essential role in the social, cognitive, and physical development of children^(3)^. However, when clinically unstable, these children reduce school attendance, which harms their biopsychosocial needs and impacts on their development, present lower performance, and face challenges in building friendships in the school setting. Therefore, a support network involving the family, school, and health professionals should be thought up, to assist them in the process of formal school education during hospitalizations due to chronic diseases^(4)^.

Teachers can play a key role in protecting these children by preventing social isolation. Health professionals can also support their development by helping schools find strategies to conciliate symptoms of illness, hospitalizations, and cognitive impairments^(5,6)^.

The bibliographic search carried out in this research on the theme of *Crianes* at school, from the perspective of the children themselves, did not find studies that show their experience in this panorama, such as the inclusion of these children in school, their perceptions regarding the disease, and their physical activity at school^(7-9)^. The research trend is focused on the family of the sick child, difficulties in home care, and educators’ management of school inclusion^(10-13)^. The studies identified gaps in terms of preparing *Crianes* for inclusion in social spaces, especially to deal with the difficulties of inclusion in the school environment, and in how these children perceive themselves in a social environment compared to other children. Recent studies indicate that there are still gaps in the integration/reintegration of *Crianes* in schools, since there is no effective communication among health professionals, the family, and the school to meet the child’s needs^(10,11)^.

Therefore, this study contributes to expand the scope of the process of caring for children with special health needs, proposing improvements in discharge plans and aiming at better insertion of children in contexts outside the hospital, in social integration, and at school. Strategies are sought for care that goes beyond the child’s clinical needs and takes their needs into account. In this context, this research had the general objective of understanding the experience of children with special health needs at school.

## METHOD

### Design of Study

Considering the unique nature of the child’s experience, the selection of qualitative research was imperative. Symbolic Interactionism^(14)^ was used as a theoretical framework and the assumptions of Grounded Theory^(15)^ as a methodological framework to understand the experience of children with special health needs at school, pointing out the meanings attributed by them to their experience in this context. The presentation of the information in this research was guided by the criteria defined by Consolidated Criteria for Reporting Qualitative Research (Coreq), used to ensure comprehensiveness, transparency, and rigor in the presentation of qualitative methods and results^(16)^.

### Local

The research was developed at the Pediatric Outpatient Clinic, which is part of the outpatient complex of a public, tertiary-level teaching and research university hospital, located in an inland city of São Paulo, Campinas, which serves an average of 32 thousand monthly consultations in 44 specialties. The outpatient clinic is in charge of providing secondary and tertiary care to children aged 0 to 18 years, operating from Monday to Friday, from 7 am to 7 pm, through pre-scheduled consultations or fitting-in, if necessary. It has a large waiting room, doctors’ offices, a nursing room, and a procedure room, where pediatric specialties (Cardiology, Pulmonology, Immunology, Nephrology, Infectology, Endocrinology, Rheumatology, Gastrology, Surgery, Neurology, Genetics, and Adolescence/Adult Transition) are provided. It is responsible for the continuity of care and treatment for children coming from inpatient units and from various municipalities in the region for monitoring chronic diseases. The hospital is a reference for the city of Campinas and for the macro-region of 86 cities that surround it. However, patients from almost all municipalities in the state of São Paulo and several Brazilian states are equally served. The pediatric outpatient nursing team consists of one nurse and three nursing technicians; the medical team includes internship students and residents, teachers, and professionals hired by the service.

### Population and Selection Criteria

The research participants were 22 school children^(17)^ with special health needs, all attending school and being followed up at the pediatric outpatient clinic of a tertiary teaching hospital.

The inclusion criterion was to be *Crianes* aged between six and 12 years old who attended school. The exclusion criteria were: *Crianes* with cognitive deficits and/or hearing impairments and children with verbal communication deficits. These were excluded because it was understood that the phenomenon of the study, in this audience, presents specificities that could not be adopted for this research due to the dynamics and time of collection, for comprehensive and inclusive communication strategies to be adopted. The sample was carried out for convenience.

### Sample Definition

Twenty-two children participated in the research. It should be highlighted that other children were invited to participate; however, it was not possible to conduct face-to-face interviews due to the pandemic scenario, which limited students and researchers’ access to the service. There were no refusals among the children invited to participate in the study. Nevertheless, there were withdrawals, as three interviews had to be interrupted when the children were called for a medical appointment, making it impossible to resume them later due to the families’ time management Chart 1 presents a description of the study participants in terms of age, diagnosis, origin, and classification.

**Chart 1:** Description of study participants. Campinas, Brazil, 2023.

Name	Age	Diagnosis	Origin	Classification CRIANES^(18)^
Blue and the fairies	11 years	Congenital megacolon	Sumaré-SP	Mixed care
Beauty and the Beast	11 years	Autoimmune Hepatitis	Iperó-SP	Medicated
Sponge Bob	7 years	Systemic Juvenile Idiopathic Arthritis	Sumaré-SP	Development demands
Snow White	12 years	Systemic Lupus Erythematosus	Campinas-SP	Medicated
Cinderella	10 years	Juvenile Dermatomyositis	Campinas-SP	Modified Habits
Joker	12 years	Tricuspid Valve Dysplasia	Hortolândia-SP	Mixed care
Dragon Ball	8 years	Atopic Dermatitis	Sumaré-SP	Medicated
Maleficent’s Daughter	10 years	Marfan syndrome	Chavantes-SP	Mixed care
Hawkeye	10 years	Myelomeningocele/Hydrocephalus	Hortolândia-SP	Mixed care
Gigi (Lucas Neto)	6 years	Congenital megacolon	Hortolândia-SP	Technological care
Spiderman	12 years	Type 1 Diabetes Mellitus	Monte Alegre do Sul-SP	Medicated
Hulk	11 years	Cystic Fibrosis	Piracicaba-SP	Mixed care
Lady Bug	9 years	Myelomeningocele/ Neurogenic Bladder/ Cystostomy	Vineyard-SP	Mixed care
Lucas Neto	8 years	Genetic Syndrome	Atibaia-SP	Mixed care
Daisy	8 years	Type 1 Diabetes Mellitus	Campinas-SP	Medicated
Messi (soccer player)	12 years	Congenital Heart Disease	Pedra Bela-SP	Mixed care
Minnie	8 years	Asthma	Monte Mor-SP	Medicated
Miriam (Biblical Cinderella Story)	8 years	Congenital Adrenal Hyperplasia	Serra Negra-SP	Development demands
Moana	11 years	AIDS	Monte Mor-SP	Medicated
Rapunzel	11 years	Asthma	Campinas-SP	Medicated
Ice Unicorn	7 years	Systemic Lupus Erythematosus/Obesity	Jarinú-SP	Mixed care
Care Bears	9 years	Asthma	Vargem Grande do Sul-SP	Medicated

### Data Collection

Data collection took place between February and March 2021, a period in which restrictions on health services due to Covid-19 were still in force, and in February 2023. The strategies used were participant observation, free drawing as an icebreaker strategy, for a subsequent semi-structured interview.

Participants were accessed in the outpatient waiting room, while they waited for medical or nursing care, accompanied by their guardians. The researcher approached both the children and their guardians in the waiting area, explaining the study, its objectives and procedures, and then invited them to participate. Throughout the development of the study, ethical aspects related to research with human beings were considered, and data collection only began after the consent of those responsible and the children, with the signing of the Free and Informed Consent Form and the Assent Form.

The participants in this study, through semi-structured interviews, discussed their experiences, before the outpatient consultation, in a private environment, located in the aforementioned outpatient clinic and previously reserved for this purpose. Families were offered the option of having the interviews take place at home or outside the hospital, but all families chose to have the interviews carried out at the outpatient clinic on the day of the child’s appointment. The responsible parents accompanied their children throughout the data collection process, from the drawing dynamic to the interview, following it fully, in common agreement with the child.

**Figure 1:**
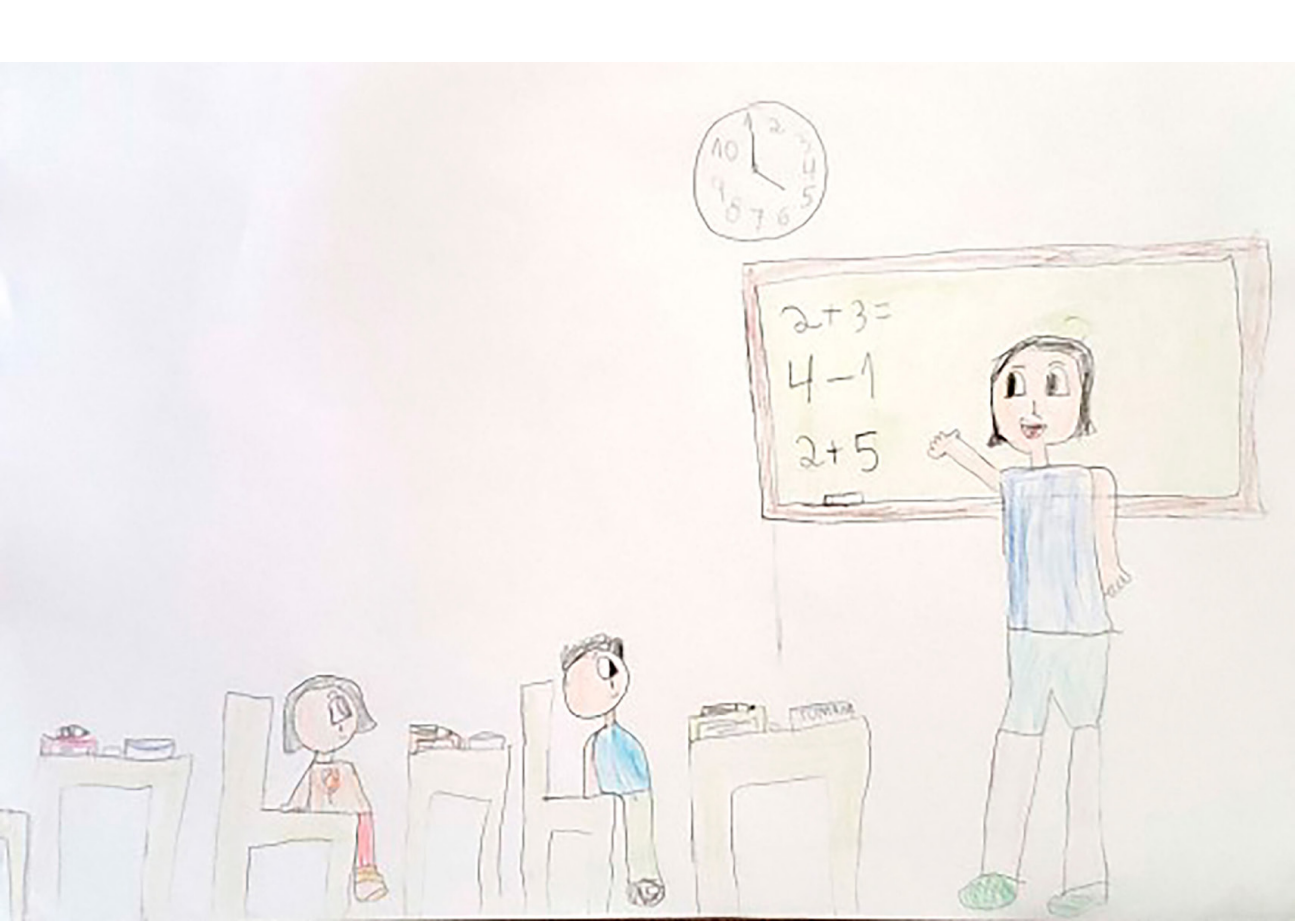
Drawing by SpongeBob, 7 years old.

**Figure 2:**
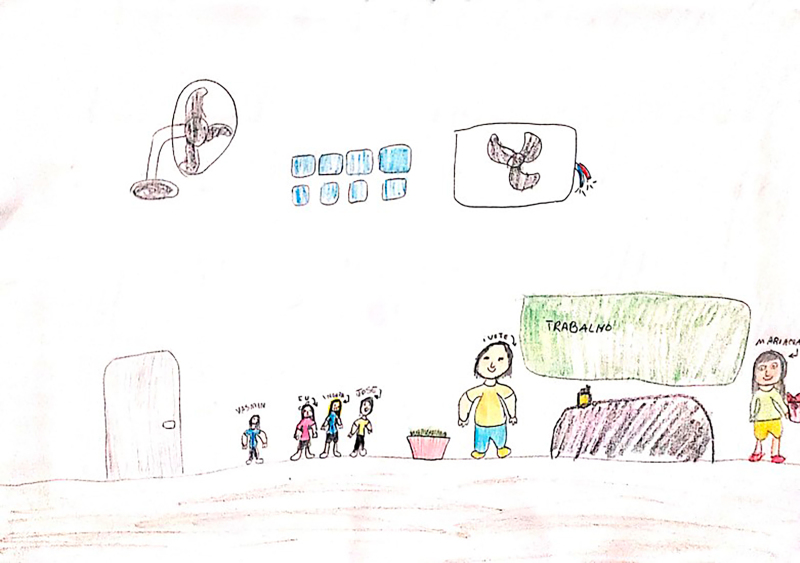
Drawing by Blue from the Fairies, 11 years old.

**Figure 3:**
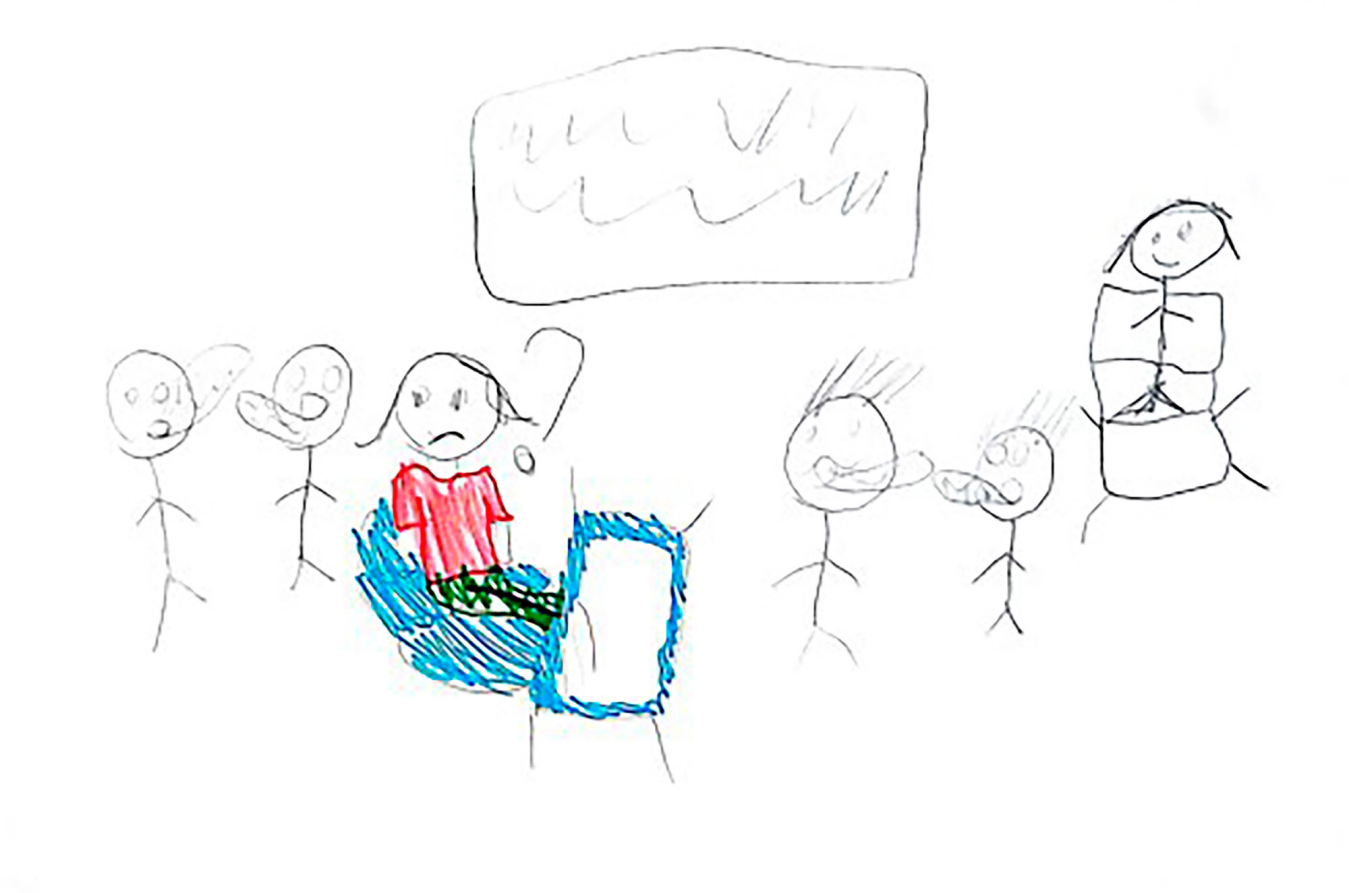
Drawing by Dragon Ball, 8 years old.

**Figure 4:**
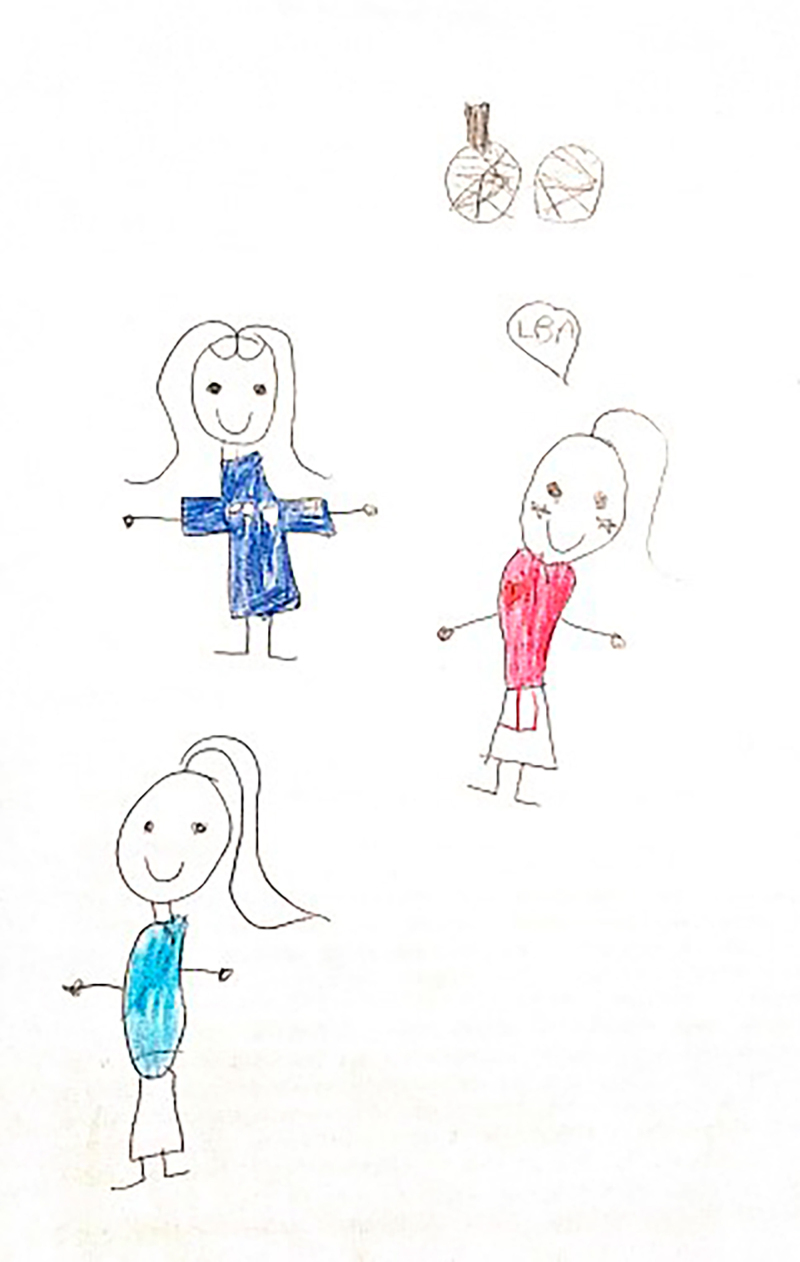
Drawing by Cinderella, 10 years old.

**Figure 5:**
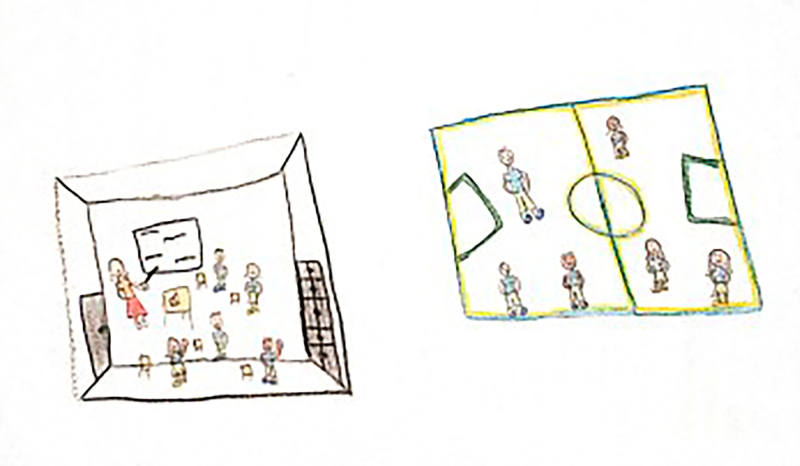
Drawing by Snow White, 12 years old.

**Figure 6:**
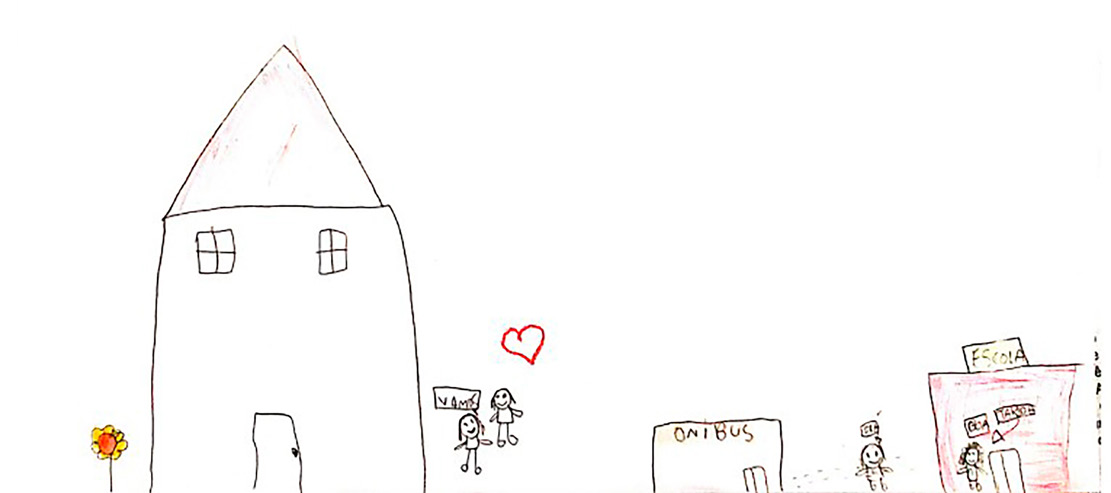
Drawing by Lady Bug, age 9.

At the beginning of the information collection, all the children stated that they enjoyed drawing, which facilitated involvement and dialogue between the researcher and the participants. Children who agreed to participate were invited to draw a picture of their daily life at school. The request was conducted through the following guiding question: “let’s draw what it’s like for you to be at school?” At that time, the researcher provided the child with the following materials: A4 sheet of paper, colored pencils, wax crayons, graphite pencil, eraser, and pencil sharpener. There were two pilot interviews and these were included as study participants. After the guiding question, the child had free time to make the drawing and, when finished, the researcher asked him/her to tell about his/her drawing or talk about what he/she drew, if he/she so wished. The researcher let the child tell his/her story, returning to the focus whenever necessary, guiding the interview with questions similar to: what was it like to have an illness and be at school? How did you feel at school compared to the other children? What was it like using your medication or device (probe, braces, wheelchair) at school? Some of the drawings made by the children are presented in the results section through Figures 1, 2, 3, 4, 5, and 6.

All interviews were recorded on digital equipment and later manually transcribed in full, without the use of software, preserving the phrases and characteristics of each child, with an average duration of 25 minutes each. Notes were made in a field diary as soon as the interviews were completed, to avoid loss and alteration of data, called “observation notes”. The transcripts were not returned to the participants for correction; however, the researcher was available for further consultation or additions if the participants deemed it necessary. Two of these conversations took place in two meetings with the child and their family member, the second being via video call to clarify specific points of the interview that raised doubts after transcription. Participants were identified by the names of children’s story characters chosen by them, ensuring confidentiality regarding their identity.

Data collection was interrupted when researchers considered the amount of information obtained sufficient to answer the research question, in addition to not presenting any new information. Data analysis was carried out simultaneously with collection, taking into account both the conceptual complexity and the stability of the codes developed, with the aim of achieving code saturation. This approach also sought to deepen the understanding of the phenomenon under study, allowing the saturation of meaning to be achieved. In this way, the reliability of the process was ensured. Furthermore, the number of participants for the research was not limited, but determined during the research, as the data were collected and analyzed^(19)^.

The collection was carried out entirely by the main researcher, with the supervision of the most experienced researcher who, at the time, did not work as a nurse at the collection site, and her employment was only as a postgraduate student in Nursing, which was explained to the research participants, as well as to their guardians. The objectives and reasons for developing the study were made clear to the participants. The researcher, who is specialized in Pediatrics, participated in training for qualitative interviews and with families in the research group in which she is involved.

It is worth noting that, initially, data collection took place at the height of the pandemic, when social isolation was in effect. Therefore, sanitary measures were adopted, such as the use of masks and hand sanitization with alcohol gel, both by the researcher and the participants, and distribution of individual kits with the materials used to make the drawing, with each child taking their own drawing home. Furthermore, the first vaccines were being administered in the country, with immunization restricted to healthcare professionals. In this context, outpatient clinics were being reopened and some schools were resuming classes; however, the majority still continued with remote teaching, so most of the participating children were not attending school in person during the data collection period.

Of the 22 participating children, 19 were in this period. For this reason, in 2023, with the pandemic under control, after children were vaccinated and returned to school, new interviews, to validate the data categories already formed, were performed. Thus, 3 new interviews were carried out, which corroborated the findings and were limited to this number, as there was no significant change in the results.

### Data Analysis and Treatment

The recorded speeches were transcribed in full and analyzed according to the constant comparative method, through which the categories were compared to identify equivalent or distinct concepts, simultaneously with data collection. Data analysis was carried out using the assumptions of Grounded Theory, which consist of open and axial coding.

The data analysis process, initially, was open coding, leading to the formation of preliminary codes, immediately after detailed analysis of the data, evaluating line by line and word by word, through the process called microanalysis. During this process, between 30 and 50 codes were listed per interview, reduced into three categories. In a second step, through a process called comparison, the codes were grouped one by one according to their conceptual similarities and differences, forming the categories^(15)^.

In this research, the type called “process” was used for axial coding. Strauss and Corbin^(18)^ define it as a series of evolutionary sequences of action/interaction that occur in time and space, changing or remaining unchanged at certain moments, in response to the situation or context. This way, the process in the data is represented by events and facts that may or may not occur in continuous sequences.

As coding and categorization occurred, questions were asked that could follow up on the data and, thus, other elements were sought until the categories became denser and theoretical saturation was achieved^(17,18)^. The themes emerged from the data analysis, which occurred entirely manually and involved two researchers at this stage.

### Ethical Aspects

For the research to be carried out, authorization was requested from the Hospital Superintendence of the aforementioned institution, and then, following the ethical precepts contained in Resolution No. 466/2012^(20)^ of the National Health Council, of the Ministry of Health (CNS/MS), the project was sent to the Research Ethics Committee (CEP) of the Universidade Estadual de Campinas, through the Plataforma Brasil, under CAAE: 33459320.1.0000.5404, being approved with Opinion number 4.137.634.

Those who agreed to participate in the study, after being invited and having their objectives explained, were asked to sign the Informed Consent Form (the child’s family member) and the Assent Form (the children and their respective guardians) based on Resolution No. 016/2000 (Art. 5, item III) of the Federal Council of Psychology. The right to refuse or withdraw was respected without any harm to the patient’s treatment.

## RESULTS

Through data analysis, it was possible to uncover and understand the experience of children with special health needs in the school environment. The meanings attributed by children to their school experience were revealed after constant analysis and comparison of the data. Thus, the process that represents the experience of children with special health needs in the school environment is made up of three categories: “Experiencing the school environment”, “Identifying obstacles”, and “Finding ways to move forward despite the illness”. Figures 1, 2, 3, 4, 5, and 6 depict some of the drawings made by the children at the beginning of their interviews.

### Experiencing the School Environment

The children’s experience in the school environment represents the child’s movement to continue developing despite their diagnosis. They recognize school as an important and necessary point in their life trajectories, a space where they share knowledge and learning, which helps in their process of building socialization, with the possibility of making new friends, allowing new discoveries. The school is incorporated as an integral part of their routine and seen as a space for interaction and play.

(...) *Very good. I like studying. Studying makes me, like, smarter, kinder, things like that.* (SpongeBob, 7 years old).

*It was cool, it was fun, then, after we studied, it was time for a break, we ate and then we played.* (...) *I remember, but I don’t know, I remember studying, it was a lot of fun! Oh yeah, it was a lot of fun.* (Gigi, 5 years old).

School is an environment that allows children to feel equal to their peers. *Experiencing the school environment* allows bringing a sense of normality into their daily lives, even if only for a few moments.

*Ah, going to school is normal for me, I think it’s quality of life for me, because when I’m at school it’s because I’m fine with my illness.* (...) *so, I think it’s normal like that, you know* (Rapunzel, 11 years old).

Children with special health needs see school as an environment of new possibilities, which gives them the opportunity to create bonds and make new friends, forming a support and safety network so that their needs are met. Along the way, they can count on friends and teachers who understand their limitations and offer support.

(...) *She told me to drink water, because I have to drink a lot of water, because I don’t drink water. Also when I was sick, she always took care of me. And gave me medicine, she came and go, told me all the time “come here”, asked me if I was in pain and measured my fever when I had a fever* (Blue of the Fairies, 11 years old).

(...) *Yes. It was the students who brought them, it wasn’t the teacher, the students would go up, because they would also get books, right, they would go up and take* (Hawkeye, 10 years old).

### Identifying Obstacles

The obstacles are varied and involve both issues related to the disease itself, which causes children to face signs and symptoms in their daily lives, and aspects linked to relationships, which may or may not be established with the groups of people with whom they live in the school environment.

The *Crianes* recognize the limitations that the disease imposes, which, in their view, can hinder their development at school and interfere with their performance. This panorama changes the way children interact with their peers and the environment in which they live. Children, through school tasks and routines, realize that they have specific needs and, also, the behavior of some classmates leads them to constantly face their limitations, generating feelings of sadness and frustration:

*One day I was in line for the bus, and the girl called me skinny* (...) *She knew that I don’t gain weight because of my illness and yet she kept calling me skinny* (...) *I don’t like it when that happens at school* (...) (Hulk, 11 years old).

Children realize that when their underlying disease becomes more severe, they face difficulties in their studies, either for keeping up with classes because they have to be absent to go to medical appointments, or for keeping up with school activities because they are not feeling well. The child understands that having an illness is a limiting factor and interferes with their development at school, especially with the worsening of the illness or due to the limitation that the illness itself brings, making social interaction with other children difficult, as they feel limited, especially by some of their classmates and teachers. The children’s speeches indicate the discomforts that arise in their daily relationships with the people they interact with at school in their daily lives, such as many people not knowing about their illness, and the contempt or lack of patience when they show their limitations.

*I couldn’t run much. Because my heart was racing and I was short of breath.* (...) *I told the director. Then she had to call my mother.* (Maleficent’s daughter, 10 years old).

*Then one day I was having a crisis, I couldn’t breathe, the director had to call my mother and we went to the hospital* (Care Bears, 9 years old).

In their narratives, the *Crianes* emphasize the displeasure caused when their colleagues, in their behavior and speech, confront them with their limitations or point them out as sick, as if the disease were a label that they must carry full time.

*Ah, when I enter the room I feel a little embarrassed, but when I spend some time there (at school) I’m not so embarrassed, because everyone (colleagues) has already asked (about the skin lesions), so I’ve already relieved a little, because no one was asking anymore, because I left, oh how embarrassing, they’re talking about me more, then it passed* (...) *Because it’s really ugly to have this here (shows the lesions on the skin), but my father and mother encourage me that this isn’t ugly, and I got used to having this and I had the strength to go to school.* (Dragon Ball, 8 years old).

The disease can impose some stigmas on the child, especially regarding their appearance, generating nicknames or fear of possible contamination. This means that the child has to constantly explain about their illness and its effects on their body, seeking to reduce stigma and foster harmonious coexistence. This is a situation that makes them sad and makes them think about dropping out of school. At these times, they feel different and excluded by their peers.

*She nodded her head (she was ashamed because of the wounds on her hands). They said they were afraid. To catch what I had!* (Cinderella, 10 years old).

*I feel good, sometimes people say I’m too short to be in the sixth grade* (Beauty and the Beast, 11 years old).

The pandemic caused by the SARS-CoV-2 virus has given children a new perspective on themselves, adding yet another challenge to this population. This episode made the *Crianes* begin to see themselves differently, as fragile beings, belonging to a risk group, a fact that was imposed on them at all times by the people around them. Covid-19 has brought changes to their life and school routine, with the suspension of in-person classes. Therefore, these children had to deal with new ways of learning, with the use of technology, bringing even more difficulties and limitations. Returning to school for them was also different, as they were the last to receive authorization to return to school.

*I can’t (go to school), I’m at a high-risk group* (SpongeBob, 7 years old).

## FINDING WAYS TO MOVE FORWARD DESPITE THE ILLNESS

Children with special health needs recognize that they have different demands compared to their peers in the school environment due to their illness, and this causes them to seek strategies to deal with the situation and be able to attend school. Showing resilience, they find ways to adapt to the situation and carry out their activities.

(...) *because I was already feeling something, then I had to stop, rest and go back, because otherwise I would be short of breath* (...) *shortness of breath, it happens quite often. Then I would stop, rest, put someone else in my place and then come back.* (Joker, 12 years old).

(...) *then I couldn’t play soccer anymore.* (...) *It’s in the stands or in the goal, before I could be the goalkeeper, after a while I couldn’t anymore* (Messi, football player, 12 years old).

The *Crianes* realize that most of their friends and school staff treat them differently and are concerned about them. Noticing this care makes them feel safe and embraced in this space, which favors their experience in this context. Embracement and safety are focused on issues involving the repercussions of their illness, the feelings of fear and insecurity that plague them when they are in the school environment.

*Oh, I think it will be normal like this, I will be able to do it, if not, my mother said that someone will help me.* (Spiderman, 12 years old) – referring to the Insulin he will have to apply at school.

One of the strategies found by *Crianes* is to recognize their limitations and focus on the care they need to take to avoid increasing or allowing the exacerbation of the symptoms of the disease. This is not an easy task for these children and it seems that it takes time to adapt so that they can have this new perception of themselves and the consequences that the disease may impose on them.

*What I did more was to play tag.* (...) *Before I didn’t feel very tired, just a little.* (...) *Little by little I saw that I had to stop* (...) *I would sit on the bench for half an hour and then I would play again.* (Hulk, 10 years old).

*Only the spots on my face meant I couldn’t stay in the sun too much in physical education class, so even so, I told the teacher, and the teacher let me sit in the shade, that’s it, but when there was a lot of sun I didn’t play as much* (Snow White, 12 years old).

In this process, some children are dependent on care that involves the use of technology, such as oxygen therapy and cystostomy catheters, which are initially performed by family members, who need to be present with the child at school to perform them, placing them in a situation of dependence on a caregiver in the school environment or available to help them if necessary. In this relationship with the disease and the need to stay in school and develop, the *Crianes* understand that, for this to happen, their independence is a fundamental factor. Thus, over time, they seek training and self-care, with the aim of achieving autonomy and independence.

*Yes, my mother was going to change the diaper* (...) *I already had a schedule* (...) *Now I’m the one who probes* (Lady Bug, 9 years old) *–* After cystostomy surgery, the child reports that she already knows how to probe, assuming self-care.

Therefore, *Finding ways to move forward despite the illness* reveals to us that the *Crianes*, despite the difficulties encountered during the process of reintegration into the school environment, are resilient and adapt to the reality they live in to feel closer to other children and continue their studies, seeking to maintain their quality of life.

## DISCUSSION

It is believed that this study contributes to bringing to light and highlighting the importance of allowing spaces for speech so that *Crianes* can report their experiences, advantages and difficulties in the process of being a child, having a special health need, and attending school. With this, it is understood that the strategy of giving voice to the child through interviews and their creativity through drawings was adequate to respond to the objective of the study. Symbolic Interactionism^(14)^ led us to think about the child interactionally and allowed us to investigate the meaning attributed by them to school, as well as their perspectives regarding the interactions established in the experiences of being a *Crianes* at school.

As well as the results of this work, previous studies, carried out at national and international levels, indicate the difficulties encountered by *Crianes* in remaining in the school environment, since signs and symptoms of the disease during treatment, physical limitations, frequent visits to medical appointments and hospitalizations are factors that directly impact the need for these children to be absent from school, hindering their learning process and, in some situations, leading to school dropout^(7,8,10,11,21,22)^. In many situations, the school routine of children with *Crianes* can be affected by the number of hospitalizations. With this, the school acquires incalculable relevance, since it will be up to it to ensure the inclusion of this student, with ethical commitment. Furthermore, quality of life and social inclusion may be present in the existence of these children and adolescents^(7)^.

Issues related to prejudice and stigma regarding some physical characteristics or limitations imposed by the disease on *Crianes* provoke unpleasant feelings and sadness, labeling them as different, abnormal and, often, leading them to believe that they may be responsible for the illness of other people. These facts are striking and decisive for the types of relationships and interactions that are formed in the school environment. All these notes are also described and discussed in previous studies that address issues involving *Crianes* and children with chronic diseases^(7,9-11)^.

When discussing the relationship between teachers and children at school, the bond is a crucial factor. Establishing bonds is essential to facilitate learning and provides a sense of satisfaction to teachers, who become more empathetic when considering the situation of the *Crianes*’ families. When educators practice empathy with those who have special health needs, they can better identify the needs, limitations, and talents of these students. This allows them to personalize teaching according to the individual characteristics of each student^(23)^, thus promoting their autonomy and participation in civic life^(24)^.

It can be stated, based on the concepts that make up Symbolic Interactionism, that *Crianes*’ behavior is influenced by the meanings they attribute to the situations they experience in their interactions with other children, health professionals and educators, with family members and with their own illness trajectory^(14)^, which can be proven with the results of this research.

Therefore, it is essential that health and education professionals receive, integrate, and include *Crianes*. This not only facilitates their participation in society, but also ensures that they receive uninterrupted assistance throughout their educational journey. Furthermore, it should be highlighted that this group of children often faces difficulties in accessing health and education services, often dealing with situations of social and economic risk^(25)^.

When discussing inclusion and accessibility in schools, it is essential to consider not only infrastructure and physical access, but also the conditions that foster student development, socialization, and learning. Investing in policies that challenge paradigms regarding the inclusion of *Crianes* is essential to overcome barriers such as intolerance, discrimination, segregation, and limiting conventions, which negatively impact the inclusion process^(9,26)^. These obstacles are frequently reflected in the statements of the children who participated in this research, highlighting the need for a broader and more conscious approach.

Specialized Educational Assistance is crucial to promoting inclusion, enabling students to develop beyond the regular school environment. This approach allows the formation of partnerships with different services, the planning of accessibility actions in educational institutions and the implementation of projects that integrate technologies appropriate for learning. For inclusive education to be successful, it is vital that there is collaboration among family, school, and a diverse network of support services, ensuring that *Crianes* are embraced and supported in all social environments^(9,10)^.

With the establishment of the Brazilian Law for the Inclusion of Persons with Disabilities (Law No. 13.146) in 2015, a new concept of total integration was established, guaranteeing the right to equal opportunities as other people when dealing with issues related to accessibility, education, and work, and to the fight against prejudice and discrimination^(27)^. However, although there has been an increase in the number of children enrolled in schools, there are still gaps to be filled, such as professionals working in primary education, who demonstrate that they do not have satisfactory knowledge about the context and do not have support networks to help include these children in school. Consequently, there is much to be developed on this issue, with public policies that really direct efforts towards improving the school inclusion of these children^(28)^, ensuring equity and respect for diversity for this specific population.

Nurses play an essential role in disseminating knowledge and promoting health education, focusing on the needs of teachers, families, children, and adolescents. In the school setting, when addressing the inclusion of *Crianes*, it is essential that the nurse dispels myths and prejudices related to insertion and the effectiveness of inclusion. Furthermore, it is important to emphasize the relevance of support networks and the need for school and family involvement in the development of these children and adolescents^(9,10)^. This aims to ensure coordinated and comprehensive assistance, guaranteeing that care is directed to the true demands of children and adolescents^(9)^.

## CONCLUSION

The study allowed us to understand the experience of the *Crianes* in the school environment through their narratives, which revealed three important points: the *Crianes* perceive the school environment as fundamental for their development and their interaction with other people, which includes other children; the disease can be a limiting factor that may interfere with their school performance; thus, they need to develop strategies to deal with the difficulties present in their trajectories to continue their academic progress. Hence, the need for acceptance, safety, and trust in the environment and in the people who make it up was identified. These are fundamental factors for the child to continue their development and learning process despite the illness.

The data collected by this study suggest possible interventions that can be carried out in this scenario, such as the need for rapprochement and joint work of the health and education sectors to integrate or reintegrate *Crianes* into the school environment, highlighting that, with technological advances in the health area, more and more *Crianes* will occupy school desks, and these places will require not only the preparation of professionals to work with these children, but also the adequate physical structure. In fact, public policies aimed at childhood need to ensure comprehensiveness and intersubjectivity in the care and assistance to this population.

As limitations of the study, it is worth noting that it cannot be carried out with all *Crianes* classification groups, which suggests expanding the sample groups for future analyses on the experiences of *Crianes*, considering forms of intervention and monitoring of these children’s health and learning.
