# Supplementary material for: The school environment in the experience of children with special health needs: a qualitative study
Source: Rev Esc Enferm USP. 2025 Jan 27;58:e20240215. doi: 10.1590/1980-220X-REEUSP-2024-0215en (PMC11771851; doi:10.1590/1980-220X-REEUSP-2024-0215en)
Supplement: Translation

# Translation: O ambiente escolar na experiência de crianças com necessidades de saúde especiais: um estudo qualitativo

**DOI:** 10.1590/1980-220X-REEUSP-2024-0215pt

**Authors:** Álida Maria de Oliveira Andreato, Eliane Tatsch Neves, Maiara Rodrigues dos Santos, Maria Angélica Marcheti, Maira Deguer Misko

**Affiliations:** 1 Universidade Estadual de Campinas, Faculdade de Enfermagem, Campinas, SP, Brasil.; 2 Universidade Federal de Santa Maria, Santa Maria, RS, Brasil.; 3 Universidade de São Paulo, Escola de Enfermagem, São Paulo, SP, Brasil.; 4 Universidade Federal de Mato Grosso do Sul, Campo Grande, MS, Brasil.

**Keywords:** Crianças com Deficiência, Inclusão Escolar, Enfermagem Pediátrica, Doença Crônica, Interacionismo Simbólico

## Abstract

**Objetivo:**

Compreender a vivência das crianças com necessidades de saúde especiais na escola.

**Método:**

Pesquisa qualitativa que empregou Interacionismo Simbólico como referencial teórico e pressupostos da Teoria Fundamentada nos Dados como referencial metodológico. Dados coletados em um ambulatório pediátrico de instituição hospitalar de ensino no interior do estado de São Paulo. Como estratégias de coleta utilizaram-se: observação participante, desenho e entrevista semiestruturada. Participaram da pesquisa 22 crianças com necessidades de saúde especiais em idade escolar, todas frequentando a escola.

**Resultados:**

Foram construídas três categorias que representam a vivência da criança com necessidades de saúde especiais na escola: Vivenciando o ambiente escolar, Identificando obstáculos e Encontrando caminhos para prosseguir apesar da doença. As vivências dessas crianças na escola permitiram compreender suas dificuldades, sua necessidade de adaptação e suas ações frente ao processo relacionado a seu desenvolvimento.

**Conclusão:**

Identificou-se a necessidade de acolhimento, segurança e confiança no ambiente escolar e nas pessoas que o compõem, que são fatores fundamentais para que a criança possa seguir seu desenvolvimento e aprendizagem, apesar da doença.

## INTRODUÇÃO

As melhorias tecnológicas e científicas, sobretudo na área da saúde, reduziram a taxa de mortalidade infantil no Brasil, provocando uma mudança epidemiológica, marcada pelo aumento dos índices de sobrevivência de crianças clinicamente frágeis. Assim, foram necessárias políticas de atenção à saúde orientadas para este grupo que demanda cuidados contínuos dos serviços de saúde, denominado Crianças com Necessidades de Saúde Especiais (Crianes), o qual inclui as crianças com condições crônicas^(1)^.

Em 2022, foi realizado o primeiro levantamento epidemiológico de prevalência de Crianes no Brasil, revelando que uma em cada quatro famílias tem uma criança de zero a 11 anos com necessidades de saúde especiais. Esse dado está associado às circunstâncias prévias de saúde da criança, à estrutura familiar e ao contexto socioeconômico e político^(2)^.

As Crianes convivem nos espaços sociais de creches e escolas, que possuem papel essencial no desenvolvimento social, cognitivo e físico da criança^(3)^. Entretanto, quando clinicamente instáveis, essas crianças têm menor frequência na escola, o que traz prejuízos às suas necessidades biopsicossociais e consequências ao seu desenvolvimento, além de apresentarem um desempenho inferior e enfrentarem desafios na construção de amizades no contexto escolar. Por isso, é preciso arquitetar uma rede de apoio envolvendo a família, profissionais da escola e da saúde, a fim de auxiliá-las no processo de formação escolar formal durante hospitalizações decorrentes das doenças crônicas^(4)^.

Os professores podem desempenhar um papel fundamental na proteção dessas crianças, evitando o afastamento social. Os profissionais de saúde também podem apoiar seu desenvolvimento, contribuindo com as escolas para identificar estratégias que conciliem sintomas de doenças, hospitalizações e deficiências cognitivas^(5,6)^.

A busca bibliográfica realizada nesta pesquisa sobre a temática de Crianes na escola, na perspectiva da própria criança, não localizou estudos que mostrem sua experiência nesse panorama, como a inclusão dessas crianças na escola, suas percepções diante da doença e atividade física delas na escola^(7-9)^. A tendência das pesquisas está focada para a família da criança doente, dificuldades do cuidado domiciliar e manejo dos educadores face à inclusão escolar^(10-13)^. Os estudos identificaram lacunas no que se refere ao preparo das Crianes para inserção nos espaços sociais, especialmente para lidar com as dificuldades da inclusão no ambiente escolar, e como essas crianças se percebem em meio a um ambiente social frente a outras crianças. Estudos recentes apontam que ainda permeiam falhas na integração/reintegração das Crianes na escola, uma vez que não há comunicação efetiva entre os profissionais de saúde, a família e a escola de modo a suprir as necessidades da criança^(10,11)^.

Diante disso, este estudo contribui para expandir a abrangência do processo de cuidar da criança com necessidade de saúde especial, propondo melhorias nos planos de alta e visando melhor inserção delas nos contextos fora do hospital, na integração social e na escola. Buscam-se estratégias para uma assistência que vá além das necessidades clínicas da criança e que leve em consideração suas necessidades. Nesse viés, esta pesquisa teve como objetivo geral compreender a vivência da criança com necessidades de saúde especiais na escola.

## MÉTODO

### Tipo do Estudo

Ao considerar o caráter único da experiência da criança, foi imperativa a escolha da pesquisa qualitativa. O Interacionismo Simbólico^(14)^ foi empregado como referencial teórico e os pressupostos da Teoria Fundamentada nos Dados^(15)^ como referencial metodológico para compreender a experiência da criança com necessidades de saúde especiais na escola, apontando os significados atribuídos por ela à sua vivência nesse contexto. A apresentação das informações desta pesquisa foi direcionada pelos critérios definidos pelo *Consolidated Criteria for Reporting Qualitative Research* (Coreq), usado para garantir a abrangência, transparência e rigor na apresentação dos métodos e resultados qualitativos^(16)^.

### Local

A pesquisa foi desenvolvida no Ambulatório de Pediatria que integra o complexo ambulatorial de um hospital universitário público, de ensino e pesquisa, de nível terciário, localizado no interior de São Paulo, no município de Campinas, que atende uma média de 32 mil consultas mensais em 44 especialidades. O ambulatório tem como atribuição realizar a atenção secundária e terciária de crianças na faixa etária de 0 a 18 anos, funcionando de segunda a sexta-feira, das 7h às 19h, por meio de consultas pré-agendadas ou encaixe, caso necessário. Conta com uma ampla sala de espera, consultórios médicos, sala de enfermagem e sala de procedimento, onde se revezam os atendimentos das especialidades pediátricas (Cardiologia, Pneumologia, Imunologia, Nefrologia, Infectologia, Endocrinologia, Reumatologia, Gastrologia, Cirurgia, Neurologia, Genética, e Adolescência/Transição Adulto). É responsável pela continuidade do atendimento e tratamento às crianças provenientes das unidades de internação e dos diversos municípios da região para seguimento de doenças crônicas. O hospital é referência para o município de Campinas e para a macrorregião de 86 municípios que estão ao seu redor. No entanto, pacientes de quase todos os municípios do estado de São Paulo e vários estados brasileiros são igualmente atendidos. A equipe de enfermagem do ambulatório pediátrico é formada por uma enfermeira e três técnicas de enfermagem, a equipe médica conta com alunos do internato e residentes, docentes e profissionais contratados pelo serviço.

### População e Critérios de Seleção

Os participantes da pesquisa foram 22 crianças em idade escolar^(17)^ com necessidades de saúde especiais, todas frequentadoras de escola e acompanhadas no ambulatório de pediatria de um hospital-escola de nível terciário.

O critério de inclusão foi ser Crianes com idade de seis a 12 anos completos que frequentassem a escola. Os critérios de exclusão foram: Crianes que apresentassem déficit cognitivo e/ou deficiência auditiva e Crianes com déficit de comunicação verbal. Estas foram excluídas por se entender que o fenômeno do estudo, nesse público, apresenta especificidades que não foram possíveis de serem adotadas para esta pesquisa pela dinâmica e tempo da coleta, para se adotar estratégias abrangentes e inclusivas de comunicação. A amostra foi efetuada por conveniência.

### Definição da Amostra

Participaram da pesquisa 22 crianças. É importante ressaltar que outras crianças foram convidadas a participar, porém, não foi possível a realização das entrevistas presenciais devido ao cenário de pandemia, que limitou o acesso de alunos e pesquisadores ao serviço. Não houve recusa entre as crianças convidadas a participar do estudo. Entretanto, houve desistências, pois três entrevistas precisaram ser interrompidas quando as crianças foram chamadas para a consulta médica, sendo impossível retomá-las posteriormente devido à organização do tempo das famílias. O Quadro 1 apresenta a descrição dos participantes do estudo quanto à idade, diagnóstico, procedência e classificação.

**Quadro 1:** Descrição dos participantes do estudo. Campinas, Brasil, 2023.

Nome	Idade	Diagnóstico	Procedência	Classificação CRIANES^(18)^
Blue e as fadinhas	11 anos	Megacólon Congênito	Sumaré-SP	Cuidados mistos
Bela e a Fera	11 anos	Hepatite Autoimune	Iperó-SP	Medicamentoso
Bob Esponja	7 anos	Artrite Idiopática Juvenil Sistêmica	Sumaré-SP	Demandas do desenvolvimento
Branca de Neve	12 anos	Lúpus Eritematoso Sistêmico	Campinas-SP	Medicamentoso
Cinderela	10 anos	Dermatomiosite Juvenil	Campinas-SP	Habituais modificados
Coringa	12 anos	Displasia Valvar Tricúspide	Hortolândia-SP	Cuidados mistos
Dragon Ball	8 anos	Dermatite Atópica	Sumaré-SP	Medicamentoso
Filha da Malévola	10 anos	Síndrome de Marfan	Chavantes-SP	Cuidados mistos
Flecha (arqueiro)	10 anos	Mielomeningocele/Hidrocefalia	Hortolândia-SP	Cuidados mistos
Gigi (Lucas Neto)	6 anos	Megacólon Congênito	Hortolândia-SP	Cuidados tecnológicos
Homem Aranha	12 anos	Diabetes Mellitus tipo 1	Monte Alegre do Sul-SP	Medicamentoso
Hulk	11 anos	Fibrose Cística	Piracicaba-SP	Cuidados mistos
Lady Bug	9 anos	Mielomeningocele/ Bexiga Neurogênica/ Cistostomia	Vinhedo-SP	Cuidados mistos
Lucas Neto	8 anos	Síndrome Genética	Atibaia-SP	Cuidados mistos
Margarida	8 anos	Diabetes Mellitus tipo 1	Campinas-SP	Medicamentoso
Messi (jogador de futebol)	12 anos	Cardiopatia Congênita	Pedra Bela-SP	Cuidados mistos
Minnie	8 anos	Asma	Monte Mor-SP	Medicamentoso
Miriam (história bíblica da Cinderela)	8 anos	Hiperplasia Adrenal Congênita	Serra Negra-SP	Demandas do desenvolvimento
Moana	11 anos	SIDA	Monte Mor-SP	Medicamentoso
Rapunzel	11 anos	Asma	Campinas-SP	Medicamentoso
Unicórnio de gelo	7 anos	Lúpus Eritematoso Sistêmico/Obesidade	Jarinú-SP	Cuidados mistos
Ursinhos carinhosos	9 anos	Asma	Vargem Grande do Sul-SP	Medicamentoso

### Coleta de Dados

A coleta de dados aconteceu entre os meses de fevereiro e março de 2021, período em que as restrições ao serviço de saúde em decorrência da Covid-19 ainda eram vigentes, e em fevereiro de 2023. As estratégias utilizadas foram observação participante, o desenho livre como estratégia de quebra-gelo, para posterior entrevista semiestruturada.

O acesso aos participantes ocorreu na sala de espera do ambulatório, enquanto aguardavam o atendimento médico ou de enfermagem, acompanhados por seus responsáveis. A pesquisadora abordou tanto as crianças quanto os responsáveis no pátio de espera, explicando a pesquisa, seus objetivos e procedimentos, e em seguida fez o convite para a participação. Durante todo o desenvolvimento do estudo, foram respeitados os aspectos éticos relacionados à pesquisa com seres humanos, e a coleta de dados só teve início após o consentimento dos responsáveis e das crianças, com a assinatura do Termo de Consentimento Livre e Esclarecido e do Termo de Assentimento.

Os participantes deste estudo, por meio de entrevistas semiestruturadas, discorreram sobre suas experiências, antes da consulta ambulatorial, em ambiente privativo, localizado no referido ambulatório e reservado previamente para tal finalidade. Foi oferecida às famílias a opção de que as entrevistas acontecessem em ambiente domiciliar ou extra-hospitalar, mas todas as famílias optaram pela realização das entrevistas nas dependências do ambulatório, no dia de consulta da criança. Os pais responsáveis acompanharam seus filhos durante todo processo de coleta dos dados, desde a dinâmica de desenho até a entrevista, acompanhando-a integralmente, em comum acordo com a criança.

No início da coleta de informações, todas as crianças afirmaram gostar de desenhar, o que facilitou o envolvimento e o diálogo entre pesquisadora e os participantes. As crianças que concordaram em participar foram convidadas a realizar um desenho que retratasse seu dia a dia na escola. A solicitação foi conduzida por meio da seguinte questão norteadora: “vamos desenhar como é para você estar na escola?”. Nesse momento, o pesquisador ofereceu à criança os seguintes materiais: folha de papel sulfite A4, lápis de cor, giz de cera, lápis grafite, borracha e apontador. Houve duas entrevistas pilotos e essas foram incluídas como participantes do estudo. Após a pergunta norteadora, a criança teve tempo livre para a confecção do desenho e, ao seu término, a pesquisadora solicitou que ela contasse a respeito de seu desenho ou falasse sobre o que desenhou, se assim o desejasse. A pesquisadora deixou a criança contar sua história, retomando o foco sempre que se fizesse necessário, guiando a entrevista, com questões semelhantes a: como era ter uma doença e estar na escola? Como você se sentia na escola em relação às outras crianças? Como era usar sua medicação ou dispositivo (sonda, órteses, cadeira de rodas) na escola? Alguns dos desenhos realizados pelas crianças são apresentados na seção dos resultados por meio das Figuras de número 1, 2,3, 4, 5 e 6.

**Figura 1:** Desenho Bob Esponja, 7 anos.

**Figura 2:** Desenho da Blue das Fadinhas, 11 anos.

**Figura 3:** Desenho do Dragon Ball, 8 anos.

**Figura 4:** Desenho da Cinderela, 10 anos.

**Figura 5:** Desenho Branca de Neve, 12 anos.

**Figura 6:** Desenho da Lady Bug, 9 anos.

Todas as entrevistas foram gravadas em equipamento digital e, posteriormente, transcritas manualmente na íntegra, sem uso de software, preservando as frases e características de cada criança, com duração média de 25 minutos cada. Foram feitas anotações, em um diário de campo, assim que as entrevistas eram encerradas, para evitar perda e alteração dos dados, denominadas de “notas de observação”. As transcrições não foram devolvidas aos participantes para correção; contudo, a pesquisadora mostrou-se disponível para posterior consulta ou adendo se os participantes julgassem necessário. Duas dessas conversas aconteceram em dois encontros com a criança e seu familiar, sendo a segunda por videochamada para elucidar pontos específicos da entrevista que geraram dúvidas após a transcrição. Os participantes foram identificados por nomes de personagens de histórias infantis escolhidos por eles, garantindo o sigilo em relação à identidade.

A coleta de dados foi interrompida quando os pesquisadores avaliaram que a quantidade de informações obtidas era suficiente para responder à pergunta de pesquisa, além de não apresentarem novidades. A análise dos dados foi realizada simultaneamente à coleta, levando em consideração tanto a complexidade conceitual quanto a estabilidade dos códigos elaborados, com o objetivo de atingir a saturação do código. Essa abordagem também buscou aprofundar a compreensão do fenômeno em estudo, permitindo alcançar a saturação de significado. Dessa forma, garantiu-se a confiabilidade do processo. Outrossim, o número de participantes para a pesquisa não foi delimitado, mas determinado no decorrer da pesquisa, à medida que os dados iam sendo coletados e analisados^(19)^.

A coleta foi feita integralmente pela pesquisadora principal, com a supervisão da pesquisadora mais experiente que, na época, não exercia a função de enfermeira no local de coleta, sendo que seu vínculo era apenas como discente da pós-graduação de Enfermagem, o que foi explicado para os participantes da pesquisa, assim como para seus responsáveis, além de se deixar claros os objetivos e razões para o desenvolvimento do estudo. A pesquisadora, com especialização na área de pediatria, participou de treinamentos para entrevistas qualitativas e com famílias junto ao grupo de pesquisa no qual está inserida.

Vale ressaltar que, em um primeiro momento, a coleta de dados ocorreu no auge da pandemia, quando o isolamento social estava em vigor. Por isso, foram adotadas medidas sanitárias, como o uso de máscara, a higienização das mãos com álcool em gel, tanto por parte da pesquisadora quanto dos participantes, e distribuição de kits individuais com os materiais utilizados para a confecção do desenho, com cada criança levando o seu para casa. Além disso, as primeiras vacinas estavam sendo aplicadas no país, com a imunização restrita aos profissionais de saúde. Neste contexto, estavam sendo reabertos os ambulatórios e algumas escolas estavam retomando as aulas; todavia, a maioria ainda permanecia com ensino remoto, por isso a maioria das crianças participantes não estava frequentando a escola de forma presencial no período da coleta de dados.

Das 22 crianças participantes, 19 estavam neste período. Por essa razão, em 2023, já com a pandemia controlada, após a vacinação das crianças e o retorno delas à escola, foram realizadas novas entrevistas, com a finalidade de validar as categorias de dados já formadas. Assim, foram realizadas 3 novas entrevistas, que corroboraram os achados e limitaram-se a este número, pois não houve mudança significativa nos resultados.

### Análise e Tratamento dos Dados

Os discursos gravados foram transcritos na íntegra e analisados de acordo com o método comparativo constante, por meio do qual as categorias foram comparadas para identificar conceitos equivalentes ou distintos, simultaneamente à coleta de dados. A análise dos dados foi realizada por meio dos pressupostos da Teoria Fundamentada nos Dados, que consistem nas codificações aberta e axial.

O processo de análise dos dados, num primeiro momento, foi a codificação aberta, levando à formação dos códigos preliminares, logo após a análise detalhada dos dados, avaliando linha por linha e palavra por palavra, por meio do processo chamado microanálise. Durante esse processo, foram elencados entre 30 a 50 códigos por entrevista, reduzidos em três categorias. Em um segundo passo, mediante um processo denominado comparação, os códigos foram agrupados um a um por suas similaridades e diferenças conceituais, formando as categorias^(15)^.

Nessa pesquisa, utilizou-se o tipo denominado “processo” para a codificação axial. Strauss e Corbin^(18)^ o definem como uma série de sequências evolutivas de ação/interação que ocorrem no tempo e espaço, mudando ou permanecendo inalteradas em alguns momentos, em resposta à situação ou ao contexto. Dessa maneira, o processo nos dados é representado por acontecimentos e fatos que podem ou não ocorrer em sequências contínuas.

Conforme ocorreram a codificação e a categorização, realizaram-se perguntas que poderiam dar sequência aos dados e, assim, outros elementos foram buscados até tornar as categorias mais densas e alcançar a saturação teórica^(17,18)^. Os temas surgiram a partir da análise dos dados que ocorreu de modo integralmente manual e envolveu duas pesquisadoras nesta etapa.

### Aspectos Éticos

Para que a pesquisa fosse realizada, foi solicitada autorização à Superintendência do Hospital da referida instituição, e após, seguindo os preceitos éticos contidos na Resolução n° 466/2012^(20)^ do Conselho Nacional de Saúde, do Ministério da Saúde (CNS/MS), o projeto foi enviado ao Comitê de Ética em Pesquisa (CEP) da Universidade Estadual de Campinas, por meio da Plataforma Brasil, sob o CAAE: 33459320.1.0000.5404, sendo aprovado sob Parecer número 4.137.634.

Aos que aceitaram participar do estudo, depois do convite e explicação de seus objetivos, solicitou-se a assinatura do Termo de Consentimento Livre Esclarecido pelo familiar da criança e do Termo de Assentimento para crianças e seus respectivos responsáveis com base na Resolução nº 016/2000 (Art. 5, item III) do Conselho Federal de Psicologia. Foi respeitado o direito à recusa ou desistência sem que houvesse qualquer prejuízo ao tratamento do paciente.

## RESULTADOS

A partir da análise dos dados, tornou-se possível desvendar e compreender a experiência da criança com necessidades de saúde especiais no ambiente escolar. Os significados atribuídos pelas crianças à sua experiência na vivência escolar foram desvelados após a análise e comparação constante dos dados. Assim, o processo que representa a vivência da criança com necessidades de saúde especiais no ambiente escolar é composto por três categorias: “Vivenciando o ambiente escolar”, “Identificando obstáculos” e “Encontrando caminhos para prosseguir apesar da doença”. As Figuras 1, 2,3,4,5 e 6 retratam alguns dos desenhos realizados pelas crianças ao início de suas entrevistas.

### Vivenciando o Ambiente Escolar

A experiência das Crianes no ambiente escolar representa o movimento da criança em seguir se desenvolvendo apesar de seu diagnóstico. Elas reconhecem na escola um ponto importante e necessário de suas trajetórias de vida, espaço onde compartilham saberes e aprendizagens, que auxilia em seu processo de construção da socialização, com a possibilidade de fazer novos amigos, viabilizando novas descobertas. A escola está incorporada como parte integrante de sua rotina e apontada como um espaço de interação e brincadeiras.

(...) *Muito bom. Eu gosto de estudar. Estudando faz eu ficar, tipo assim, mais esperto, mais gentil, essas coisas assim* (Bob Esponja, 7 anos).

*Era legal, era divertido, depois, depois que a gente estudava, chegava a hora do recreio, a gente comia para depois a gente brincar.* (...) *Eu lembro, mas não sei, eu lembro de estudar, era muito divertido! Ah, é, era muito divertido* (Gigi, 5 anos).

A escola é um ambiente que propicia às crianças se sentirem como iguais aos seus colegas. *Vivenciando o ambiente escolar* permite trazer o senso de normalidade para seu cotidiano, mesmo que por momentos pontuais.

*Ah, ir na escola para mim é normal, eu acho que para mim é qualidade de vida, porque quando estou na escola é porque eu estou bem da minha doença.* (...) *então, eu acho que é normal assim sabe* (Rapunzel, 11 anos).

A criança com necessidade de saúde especial enxerga na escola um ambiente de novas possibilidades, que a oportuniza criar vínculos e fazer novas amizades, formando uma rede de apoio e segurança para que suas necessidades sejam atendidas. Nesse percurso, contam com amigos e professores que entendem suas limitações e oferecem apoio e suporte.

(...) *Ela falava para eu beber água, porque eu tenho que beber bastante água, porque eu não bebo água. Também quando eu estava doente, ela sempre cuidava de mim. E dá remédio, então, ia lá e ficava toda hora vem aqui, falava se estava com dor e media a minha febre quando eu tivesse com febre* (Blue das Fadinhas, 11 anos).

(...) *É. Os alunos que traziam, não era a professora, os alunos iam subir, porque eles também iam pegar livro, né, eles iam subir e pegavam* (Flecha, 10 anos).

### Identificando Obstáculos

Os obstáculos são variados e envolvem tanto questões atinentes à própria doença, que faz com que as crianças enfrentem sinais e sintomas no seu dia a dia, quanto aspectos ligados às relações, que podem ou não ser estabelecidas com os grupos de pessoas com quem convivem no ambiente escolar.

As Crianes reconhecem as limitações que a doença impõe, o que, em suas visões, pode dificultar seu desenvolvimento na escola e interferir em seu aproveitamento. Esse panorama altera a forma como a criança interage com os seus pares e com o meio no qual convive. A criança, por meio de tarefas e rotinas escolares, percebe que tem necessidades específicas e também o comportamento de alguns colegas leva-a a defrontar com suas limitações constantemente, gerando sentimento de tristeza e frustração:

*Que um dia eu estava na fila para o ônibus, aí a menina xingou eu de magrelo* (...) *Ela sabia que eu não engordo por causa da minha doença e mesmo assim ficava me xingando de magrelo* (...) *eu não gosto quando acontece isso na escola* (...) (Hulk, 11 anos).

As crianças percebem que, quando há exacerbação ou agudização da sua doença de base, enfrentam dificuldades nos estudos, seja para acompanhar as aulas, porque precisam se ausentar para ir às consultas médicas, ou para acompanhar as atividades escolares, por não se sentirem bem. A criança entende que ter uma doença é um fator limitante e interfere no seu desenvolvimento na escola, principalmente com a agudização da doença ou pela própria limitação que a doença traz, dificultando a interação social com as outras crianças, pois se sente limitada, sobretudo por alguns de seus colegas e professores. Os discursos das crianças sinalizam os incômodos que surgem nas relações diárias com as pessoas com as quais convivem na escola em seu dia a dia, como o desconhecimento de muitos de sua doença, o desprezo ou a falta de paciência quando apresentam suas limitações.

*Eu não podia correr muito. Porque meu coração ficava acelerado e eu ficava com falta de ar.* (...) *Falei para diretora. Aí teve que ligar para minha mãe* (Filha da Malévola, 10 anos).

*Ai um dia eu estava com a crise, eu não conseguia respirar, a diretora teve que chamar a minha mãe e a gente foi para o hospital* (Ursinhos Carinhosos, 9 anos).

Em suas narrativas, as Crianes colocam com ênfase o desagrado causado quando seus colegas, em seus comportamentos e falas, os confrontam com suas limitações ou os apontam como os doentes, como se a doença fosse um rótulo que devem carregar em tempo integral.

*Ah, na hora que entro na sala fico meio envergonhado, mas quando eu passo um tempo lá (na escola) eu não fico com tanta vergonha, porque todo mundo (colegas) já perguntou (sobre as lesões na pele), aí eu já aliviei um pouco, porque ninguém estava mais perguntando, porque eu saí, ai que vergonha, eles estão mais falando de mim, aí passou* (...) *Porque é muito feio ficar com isso aqui (mostra as lesões na pele), mas meu pai e minha mãe incentiva que isso não é feio, e eu acostumei a ficar com isso e eu tive força para ir para escola* (Dragon Ball, 8 anos).

A doença pode impor à criança alguns estigmas, principalmente no tocante à sua aparência, gerando apelidos ou o medo de possíveis contaminações. Isso faz com que a criança tenha que explicar o tempo todo sobre sua doença e os efeitos em seu corpo, buscando diminuir estigmas e uma convivência harmoniosa. Essa é uma situação que a deixa triste e a faz pensar em desistir da escola. Nesses momentos, sente-se diferente e excluída pelos colegas.

*Afirma que sim com a cabeça (sentia vergonha por causa das feridas nas mãos). Elas disseram assim que tinham medo. De pegar o que eu tinha!* (Cinderela, 10 anos).

*Me sinto bem, às vezes as pessoas falam que sou muito baixinha para estar no sexto ano* (Bela e a Fera, 11 anos).

A pandemia ocasionada pelo vírus SARS-CoV-2 colocou a criança frente a um novo olhar sobre si mesma, somando mais um desafio para essa população. Esse episódio fez com que as Crianes passassem a se ver diferentes, como um ser frágil, pertencente a um grupo de risco, fato que lhe era imposto a todo momento pelas pessoas em seu entorno. A Covid-19 trouxe alterações à vida e à sua rotina escolar, com a suspensão das aulas presenciais. Dessa forma, estas crianças precisaram conviver com novas maneiras de aprender, com o uso da tecnologia, trazendo ainda mais dificuldades e limitações. O retorno à escola para elas também foi diferente, uma vez que foram as últimas a receberem autorização para voltarem a frequentar o ambiente escolar.

*Não (posso ir à escola), sou da área de risco* (Bob Esponja, 7 anos).

## ENCONTRANDO CAMINHOS PARA PROSSEGUIR APESAR DA DOENÇA

A criança com necessidades de saúde especiais reconhece ter demandas diferentes de seus colegas no ambiente escolar em decorrência de sua doença, e isso faz com que busque estratégias para lidar com a situação e conseguir frequentar a escola. Mostrando-se resiliente, ela encontra caminhos para se adaptar à situação e realizar suas atividades.

(...) *porque eu já estava sentindo uma coisa, aí eu tinha que parar, descansar e voltar, porque senão eu ficava com falta de ar* (...) *falta de ar, dá bastante vezes. Aí eu parava, descansava, colocava outro no meu lugar e depois voltava* (Coringa, 12 anos).

(...) *aí eu não podia mais jogar futebol.* (...) É na arquibancada ou no gol, antes eu podia no gol, depois de um tempo eu não podia mais (Messi, jogador de futebol, 12 anos).

As Crianes percebem que a maioria dos amigos e funcionários da escola tratam-na de forma diferenciada e preocupam-se com ela. Perceber este cuidado faz com que se sintam seguras e acolhidas nesse espaço, o que favorece sua experiência nesse contexto. O acolhimento e a segurança estão voltados para as questões que envolvem as repercussões de sua doença, os sentimentos de medo e insegurança que a assolam quando estão no ambiente escolar.

*Ah eu acho que vai ser normal assim, eu vou conseguir fazer, se não minha mãe disse que alguém irá me ajudar* (Homem Aranha, 12 anos) - referindo-se à Insulina que terá que aplicar na escola.

Uma das estratégias encontradas pelas Crianes é reconhecer suas limitações e focar nos cuidados que precisam ter para não aumentar ou permitir a exacerbação dos sintomas da doença. Esta não é uma tarefa fácil para estas crianças e parece ser necessário um tempo de adaptação para que consigam ter essa nova percepção de si próprias e das consequências que a doença lhes possa impor.

*Eu brincava mais é de pega-pega.* (...) *Antes não sentia muito cansado, bem pouquinho.* (...) *Aos poucos fui vendo que tinha que parar* (...) *Eu sentava no banco e ficava uma meia horinha e depois eu brincava de novo* (Hulk, 10 anos).

*Só as manchas no rosto que eu não podia ficar muito no sol na aula de educação física, daí, mesmo assim, eu falava pro professor, aí o professor deixava eu ficar sentada na sombra, aí só isso, mas quando tinha muito sol eu não brincava tanto* (Branca de Neve, 12 anos).

Nesse processo, algumas crianças são dependentes de cuidados que envolvem o uso de tecnologia, como oxigenoterapia e cateteres de cistostomia que, de início, são realizados por familiares, que precisam estar presentes com a criança na escola para realizá-los, colocando-os em uma situação de dependência de um cuidador no ambiente escolar ou disponível para auxiliá-lo em caso de necessidade. Nessa relação com a doença e a necessidade de se manter na escola e se desenvolver, as Crianes entendem que, para que isso ocorra, sua independência é um fator fundamental. Assim, com o passar do tempo, buscam por sua capacitação e autocuidado, no intuito de conquistar sua autonomia e independência.

*Sim, minha mãe que ia trocar a fralda* (...) *Já tinha um horário* (...) *Agora eu que sondo* (Lady Bug, 9 anos) *–* após a cirurgia de cistostomia, a criança refere que já sabe sondar, assumindo o autocuidado.

Assim, *Encontrando caminhos para prosseguir apesar da doença* nos revela que as Crianes, apesar das dificuldades encontradas durante o processo de reinserção no ambiente escolar, são resilientes e adaptam-se à realidade que vivem para se sentirem mais próximas das outras crianças e darem continuidade aos estudos, buscando manter sua qualidade de vida.

## DISCUSSÃO

Acredita-se que este estudo contribui no sentido de trazer à tona e destacar a importância de se permitir espaços de fala para que as Crianes relatem suas experiências, facilidades e dificuldades frente ao processo de ser uma criança, ter uma necessidade de saúde especial e frequentar a escola. Com isso, compreende-se que a estratégia de dar voz à criança por meio das entrevistas e à sua criatividade pelos desenhos foi adequada para responder ao objetivo do estudo. O Interacionismo Simbólico^(14)^ nos levou a pensar a criança interacionalmente e nos permitiu investigar o significado atribuído por ela à escola, bem como suas perspectivas frente às interações estabelecidas nas vivências em ser uma Crianes na escola.

Assim como os resultados deste trabalho, estudos anteriores, realizados em âmbito nacional e internacional, sinalizam as dificuldades encontradas pelas Crianes para se manterem no ambiente escolar, posto que sinais e sintomas da doença ao tratamento, limitações físicas, idas frequentes às consultas médicas e hospitalizações são fatores que impactam diretamente na necessidade de que essas crianças se ausentem da escola, dificultando seu processo de aprendizagem e, em algumas situações, levando ao abandono escolar^(7,8,10,11,21,22)^. Em muitas situações, a rotina escolar das Crianes pode ser afetada pela soma de hospitalizações. Com isso, a escola adquire relevância incalculável, visto que caberá a ela zelar pela inclusão desse aluno, com compromisso ético. Ademais, a qualidade de vida e a inserção social poderão se fazer presentes na existência dessas crianças e adolescentes^(7)^.

As questões relacionadas ao preconceito e estigma frente a algumas características físicas ou limitações impostas pela doença às Crianes provocam sentimentos desagradáveis e de tristeza, rotulando-as como diferentes, anormais e, muitas vezes, levando-as a acreditar que podem ser responsáveis pelo adoecimento de outras pessoas. Esses fatos são marcantes e decisivos para os tipos de relacionamentos e interações que são formados no ambiente escolar. Todos esses apontamentos são também descritos e discutidos em estudos anteriores que abordam as questões que envolvem Crianes e crianças com doenças crônicas^(7,9-11)^.

Ao discorrer acerca da relação professor e Crianes na escola, o vínculo é um fator crucial. Estabelecer vínculos é essencial para facilitar o aprendizado e proporciona uma sensação de satisfação aos professores, que se tornam mais empáticos ao considerar a situação das famílias das Crianes. Quando os educadores praticam a empatia com aqueles que têm necessidades de saúde especiais, eles podem identificar melhor as demandas, limitações e talentos desses alunos. Isso lhes permite personalizar o ensino de acordo com as características individuais de cada estudante^(23)^, promovendo assim sua autonomia e sua participação na vida cidadã^(24)^.

Pode-se afirmar, a partir dos conceitos que compõem o Interacionismo Simbólico, que o comportamento das Crianes recebe influência dos significados por ela atribuídos às situações que vivencia na interação com outras crianças, profissionais da área da saúde e educadores, com familiares e com sua própria trajetória de doença^(14)^, o que pode ser comprovado com os resultados desta pesquisa.

Portanto, é fundamental que os profissionais da saúde e da educação recebam, integrem e incluam as Crianes. Isso não apenas facilita sua participação na sociedade, mas também garante que recebam assistência ininterrupta ao longo de sua trajetória escolar. Outrossim, é importante destacar que esse grupo de crianças frequentemente enfrenta dificuldades para acessar serviços de saúde e educação, muitas vezes lidando com situações de risco social e econômico^(25)^.

Ao discutir a inclusão e a acessibilidade nas escolas, é fundamental considerar não apenas a infraestrutura e o acesso físico, mas também as condições que propiciam o desenvolvimento, a socialização e a aprendizagem dos alunos. Investir em políticas que desafiem os paradigmas sobre a inserção de Crianes é essencial para superar barreiras como intolerância, discriminação, segregação e convecções limitantes, que impactam negativamente o processo de inclusão^(9,26)^. Esses obstáculos são frequentemente refletidos nas falas das crianças que participaram desta pesquisa, evidenciando a necessidade de uma abordagem mais ampla e consciente.

O Atendimento Educacional Especializado é crucial para promover a inclusão, possibilitando que os alunos se desenvolvam além do ambiente escolar regular. Essa abordagem permite a formação de parcerias com diferentes serviços, o planejamento de ações de acessibilidade nas instituições de ensino e a implementação de projetos que integrem tecnologias adequadas ao aprendizado. Para que a educação inclusiva tenha sucesso, é vital que haja uma colaboração entre família, escola e uma rede diversificada de serviços de apoio, garantindo que as Crianes sejam bem acolhidas e apoiadas em todos os espaços sociais^(9,10)^.

Com a instituição da Lei Brasileira de Inclusão da Pessoa com Deficiência (Lei nº 13.146) em 2015, estabeleceu-se um novo conceito de integração total, garantindo direito à igualdade de oportunidades com as outras pessoas ao tratar de questões pertinentes à acessibilidade, educação e trabalho e ao combate ao preconceito e à discriminação^(27)^. Contudo, embora tenha tido um aumento do número de matriculados nas escolas, permanecem lacunas a serem preenchidas, como os profissionais que trabalham no ensino infantil, que demonstram não ter conhecimento satisfatório sobre o contexto e não contam com redes de apoio para auxiliar na inclusão dessas crianças na escola. Assim, há muito que se desenvolver nessa questão, com políticas públicas que realmente direcionem esforços para a melhora da inclusão escolar dessas crianças^(28)^ com garantia da equidade e do respeito à diversidade para esta população específica.

O enfermeiro desempenha um papel essencial na disseminação de conhecimentos e na promoção da educação em saúde, com foco nas necessidades de professores, famílias, crianças e adolescentes. No contexto escolar, ao abordar a inclusão das Crianes, é fundamental que o enfermeiro desfaça mitos e preconceitos relacionados à inserção e à eficácia da inclusão. Além disso, é importante enfatizar a relevância das redes de apoio e a necessidade de envolvimento da escola e da família no desenvolvimento dessas crianças e adolescentes^(9,10)^. Isso visa garantir uma assistência coordenada e abrangente, assegurando que os cuidados sejam direcionados às verdadeiras demandas das crianças e adolescentes^(9)^.

## CONCLUSÃO

A pesquisa permitiu compreender a vivência da Crianes no ambiente escolar por meio de suas narrativas que desvelaram três pontos importantes: a Crianes percebe o ambiente escolar como fundamental para o seu desenvolvimento e sua interação com outras pessoas, o que inclui outras crianças; a doença pode ser um fator limitante que poderá interferir no seu desempenho escolar; a partir disso, precisam desenvolver estratégias para lidarem com as dificuldades presentes em suas trajetórias para continuar seu progresso escolar. Assim, identificou-se a necessidade de acolhimento, segurança e confiança no ambiente e nas pessoas que o compõem. Esses são fatores fundamentais para que a criança possa seguir seu processo de desenvolvimento e aprendizagem apesar da doença.

Os dados levantados pelo presente estudo propiciam sugerir possíveis intervenções que podem ser realizadas neste cenário, como a necessidade de aproximação e trabalho conjunto entre os setores da saúde e educação para integração ou reintegração das Crianes no ambiente escolar, ressaltando que, com os avanços tecnológicos na área da saúde, cada vez mais Crianes ocuparão os bancos escolares, e esses locais, além do preparo dos profissionais para trabalharem com essas crianças, precisam estar também adequados em sua estrutura física. As políticas públicas voltadas para a infância precisam, de fato, garantir a integralidade e a intersubjetividade no cuidado e assistência a esta população.

Como limitações do estudo, pontua-se que não pode ser realizado com todos os grupos de classificação de Crianes, o que faz sugerir a ampliação dos grupos amostrais para análises futuras sobre as experiências das Crianes, pensando-se em formas de intervenção e acompanhamento de saúde e de aprendizagem dessas crianças.
